# Supplementary material for: On the post-glacial spread of human commensal Arabidopsis thaliana
Source: Nat Commun. 2017 Feb 9;8:14458. doi: 10.1038/ncomms14458 (PMC5309843; doi:10.1038/ncomms14458)
Supplement: Supplementary Information — Supplementary Figures [file ncomms14458-s1.pdf]

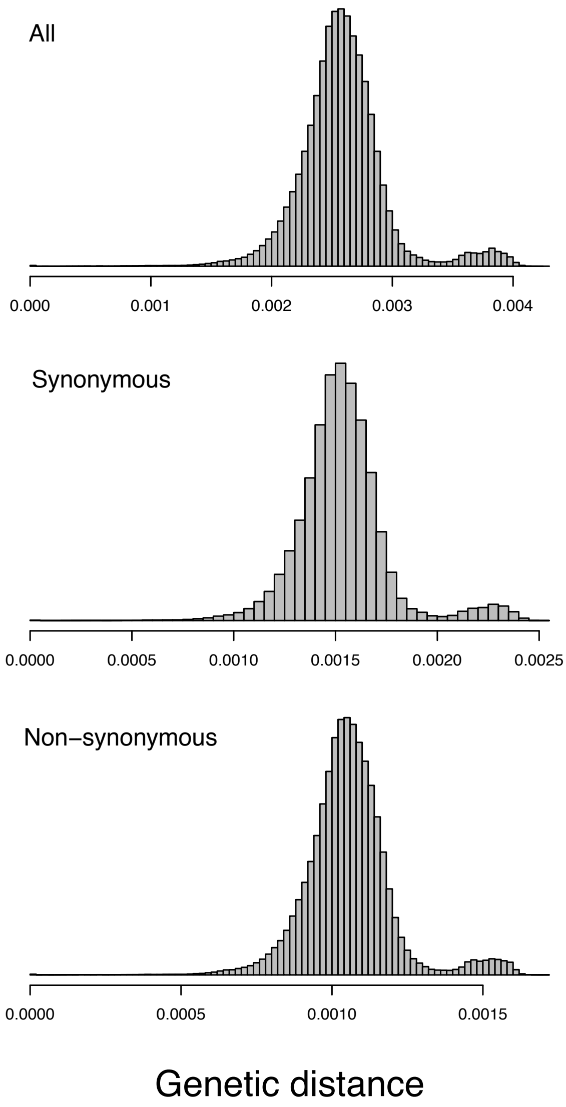

Supplementary Figure 1. Pairwise genomic distances of the 1002 accessions from Eurasia and northern Africa.

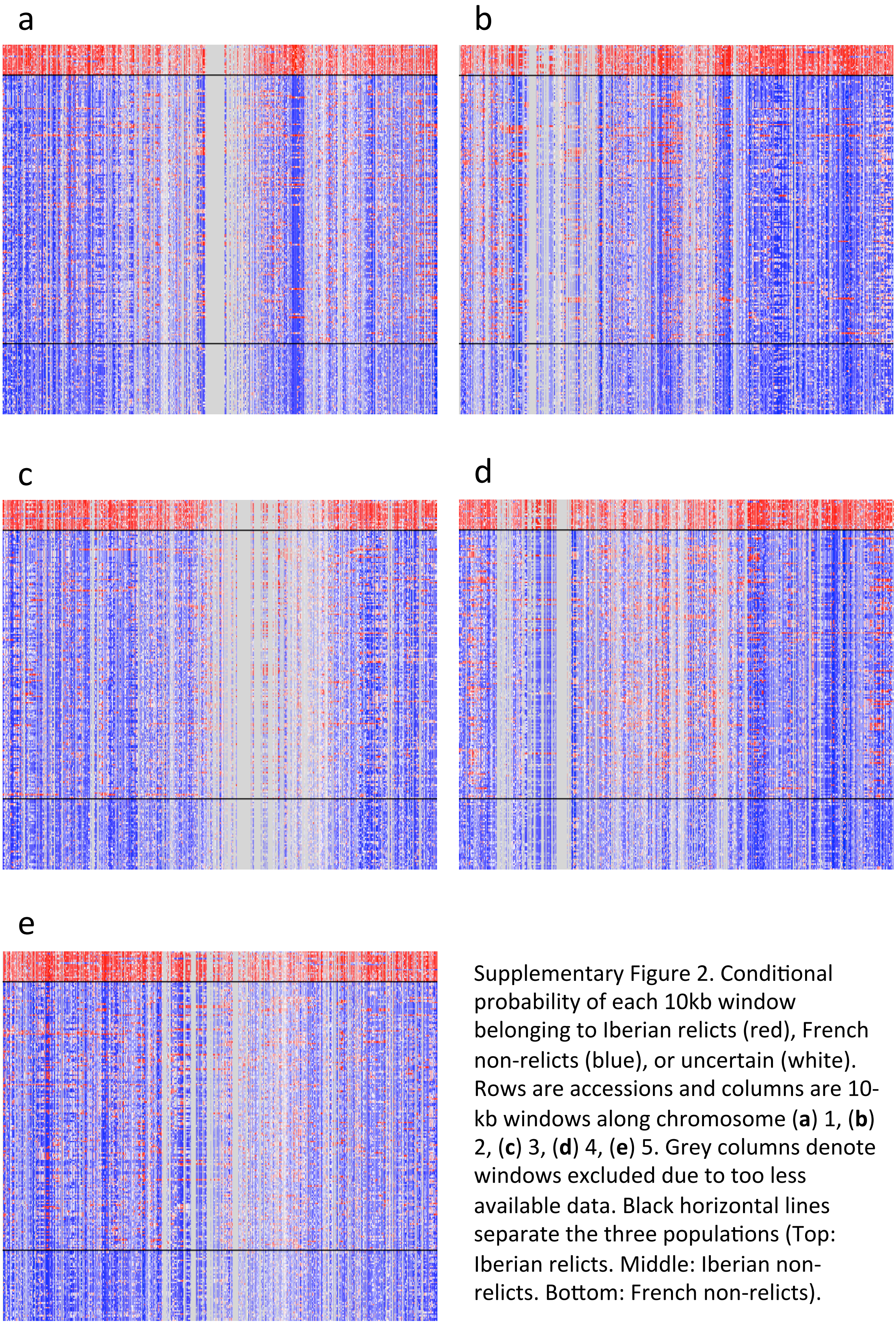

Supplementary Figure 2. Conditional probability of each 10kb window belonging to Iberian relicts (red), French non-relicts (blue), or uncertain (white). Rows are accessions and columns are 10-kb windows along chromosome (**a**) 1, (**b**) 2, (**c**) 3, (**d**) 4, (**e**) 5. Grey columns denote windows excluded due to too less available data. Black horizontal lines separate the three populations (Top: Iberian relicts. Middle: Iberian non-relicts. Bottom: French non-relicts).

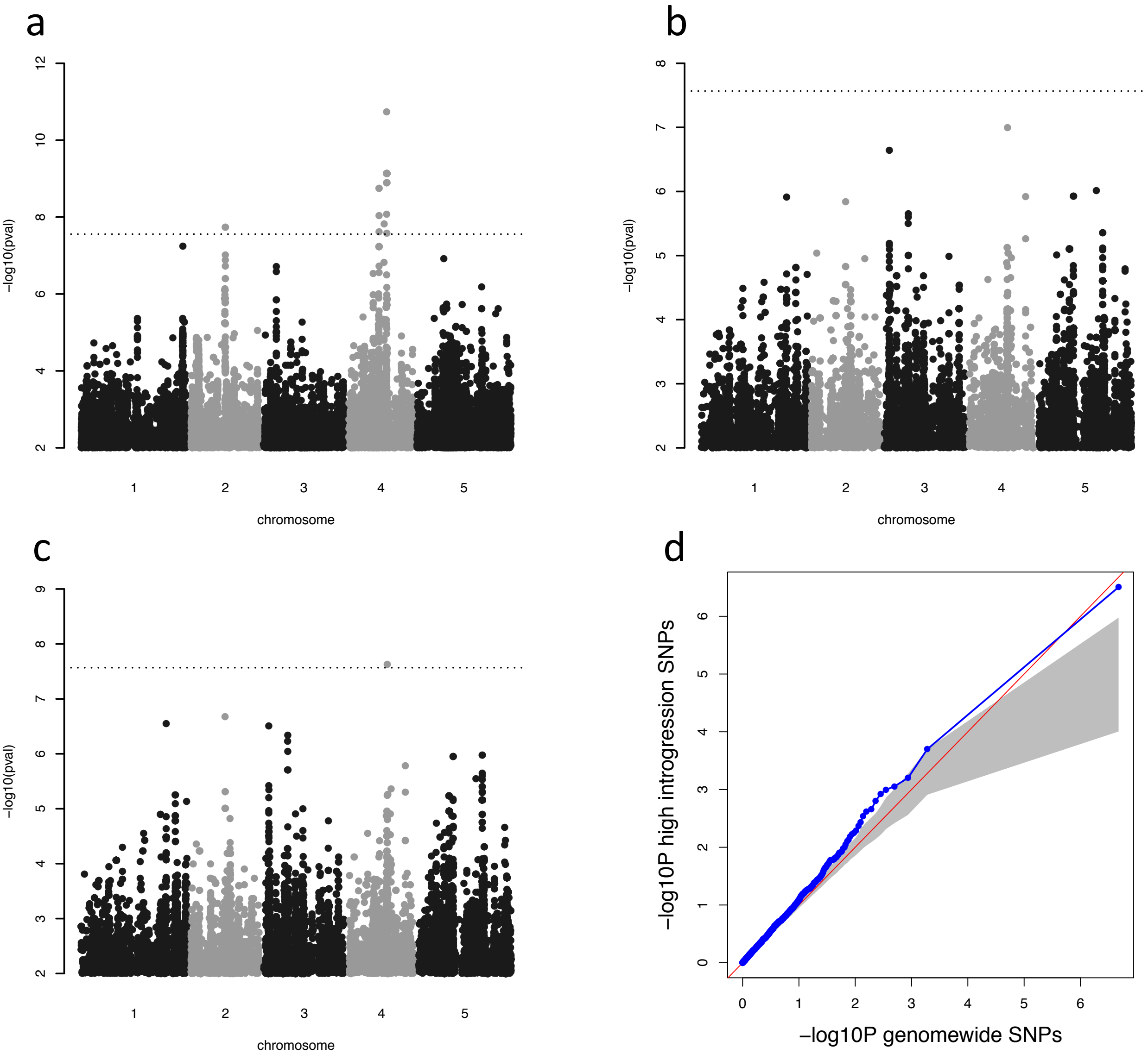

Supplementary Figure 3. Genome-wide association study (GWAS) results of (**a**) climate PC2 among all Eurasian non-relicts, (**b**) flowering time and (**c**) leaf number when flowering in 115 Iberian non-relicts. All three analyses identified the same SNP (chr4: 10,999,188) with highest score. (**d**) QQ-plots showing the enrichment of high leaf-number-when-flowering GWAS scores of SNPs with high Iberian relict introgression into Iberian non-relicts versus genomic SNPs with similar allele frequencies. This analysis excludes the 1Mb region near SNP chr4:10999188. Blue dots and line represent true value distribution, and the grey area denotes 5% significance thresholds based on 1,000 permutations.

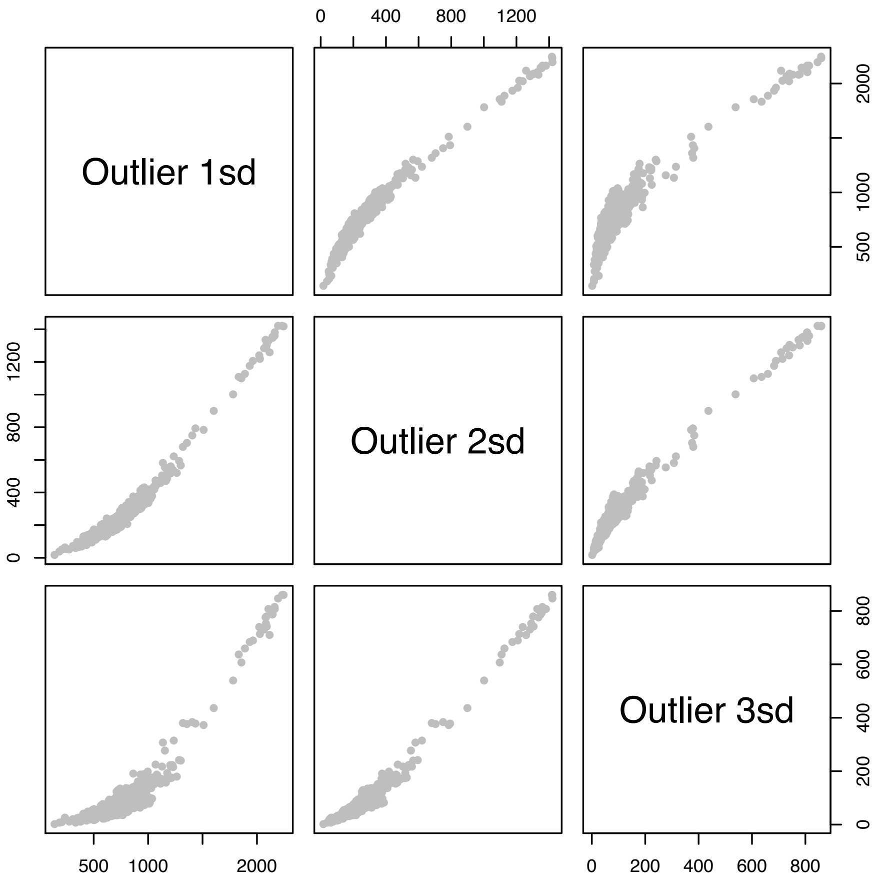

Supplementary Figure 4. Strong correlation of number of outlier windows in each accession, identified either by the 1sd (one standard deviation), 2sd, or 3sd criteria.

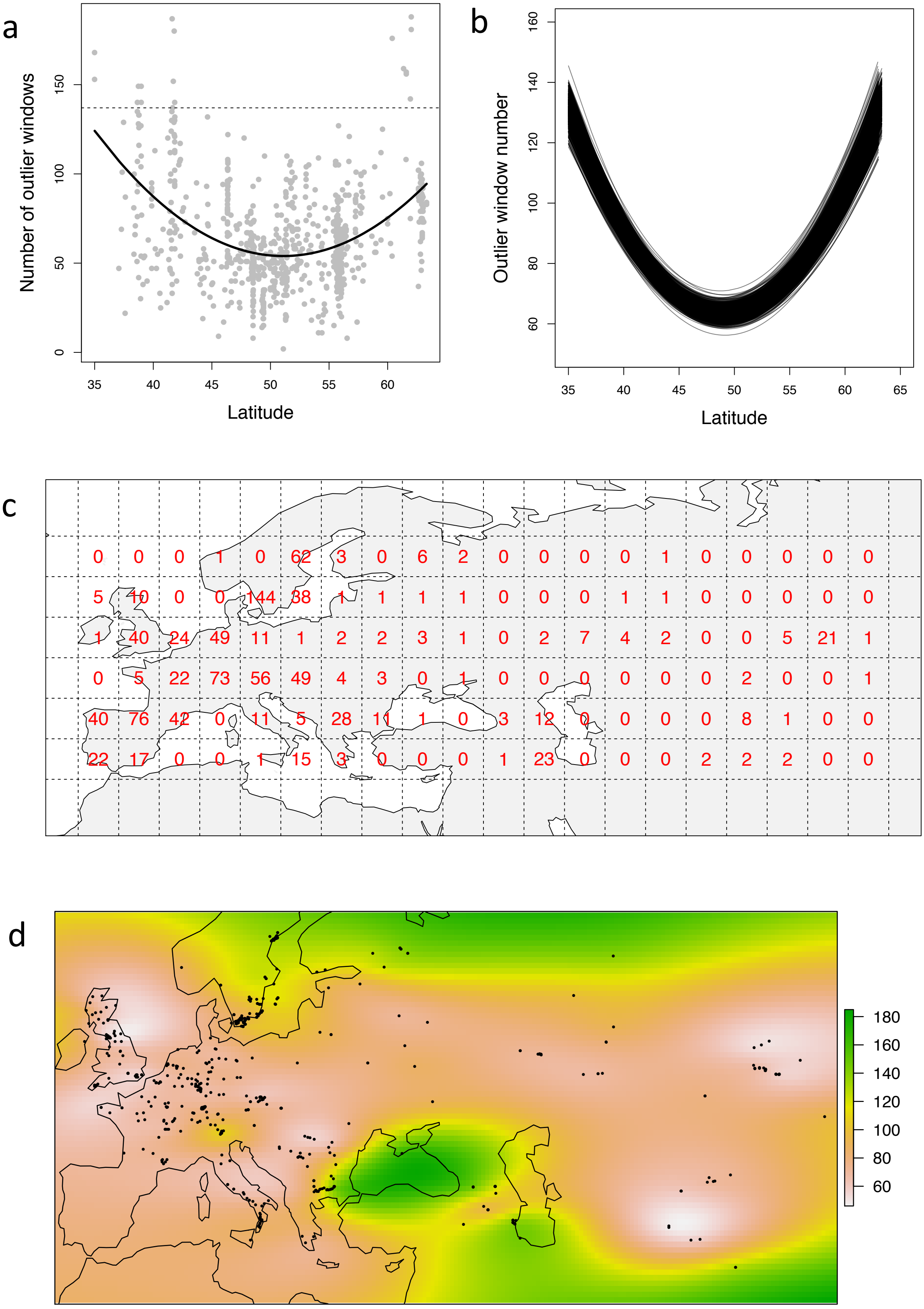

a
Number of outlier windows
0
50
100
150
35
40
45
50
55
60
Latitude
b
Outlier window number
60
80
100
120
140
160
35
40
45
50
55
60
65
Latitude
c
0 0 0 1 0 62 3 0 6 2 0 0 0 0 1 0 0 0 0 0
5 10 0 0 144 38 1 1 1 1 0 0 0 1 1 0 0 0 0 0
1 40 24 49 11 1 2 2 3 1 0 2 7 4 2 0 0 5 21 1
0 5 22 73 56 49 4 3 0 1 0 0 0 0 0 0 2 0 0 1
40 76 42 0 11 5 28 11 1 0 3 12 0 0 0 0 8 1 0 0
22 17 0 0 1 15 3 0 0 0 1 23 0 0 0 2 2 2 0 0
d
180
160
140
120
100
80
60

e

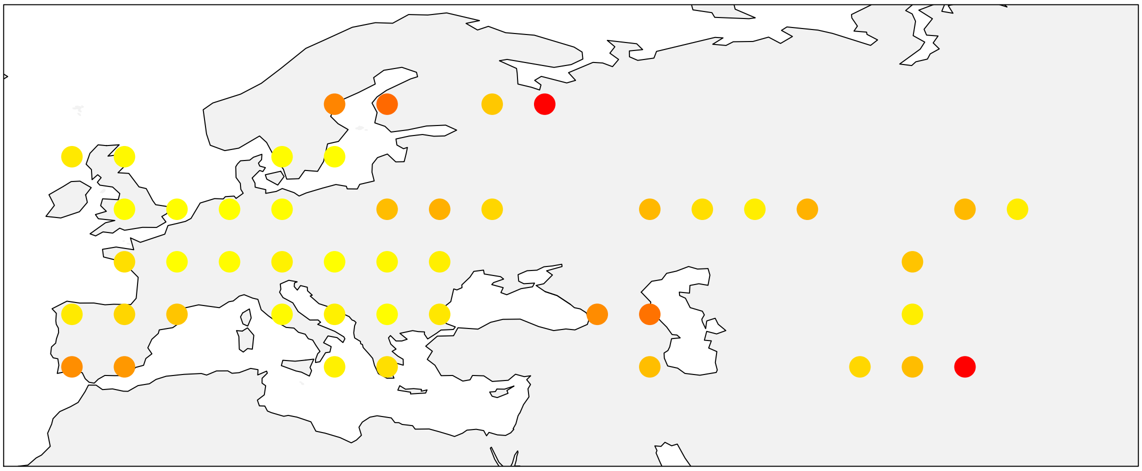

Supplementary Figure 5. Original (**a**), re-sampling (**b-d**) and grid-based (**e**) investigation of the geographic patterns of outlier haplotypes. (**a**) Excluding relicts and Iberian non-relicts, accessions in the north and south of species range are still enriched for outlier haplotypes. (**b**) In each grid of 5x5 longitude by latitude degrees, we randomly sampled 10 accessions if there are more than 10 and performed the outlier haplotype analysis again. Each line in this graph represents the result from one resampling trial. This graph contains 1,000 lines. (**c**) Grids for resampling and original number of accessions in each grid. (**d**) Resampling analyses using only synonymous distances, with number of outlier haplotypes interpolated on the map. In (**b**) and (**d**), all accessions were analyzed, but relicts and Iberian non-relicts were excluded in plotting due to the exceptionally high amount of outlier haplotypes. (**e**) Geographic-grid-based outlier haplotype analyses. Instead of resampling individuals, we treated accessions within each geographical grid as a population and identified outlier haplotypes by comparing population, rather than individual, distances in each 10kb window. Yellow represents less and red represents more outlier haplotypes. The maps were created with data from package “rworldmap” of R.

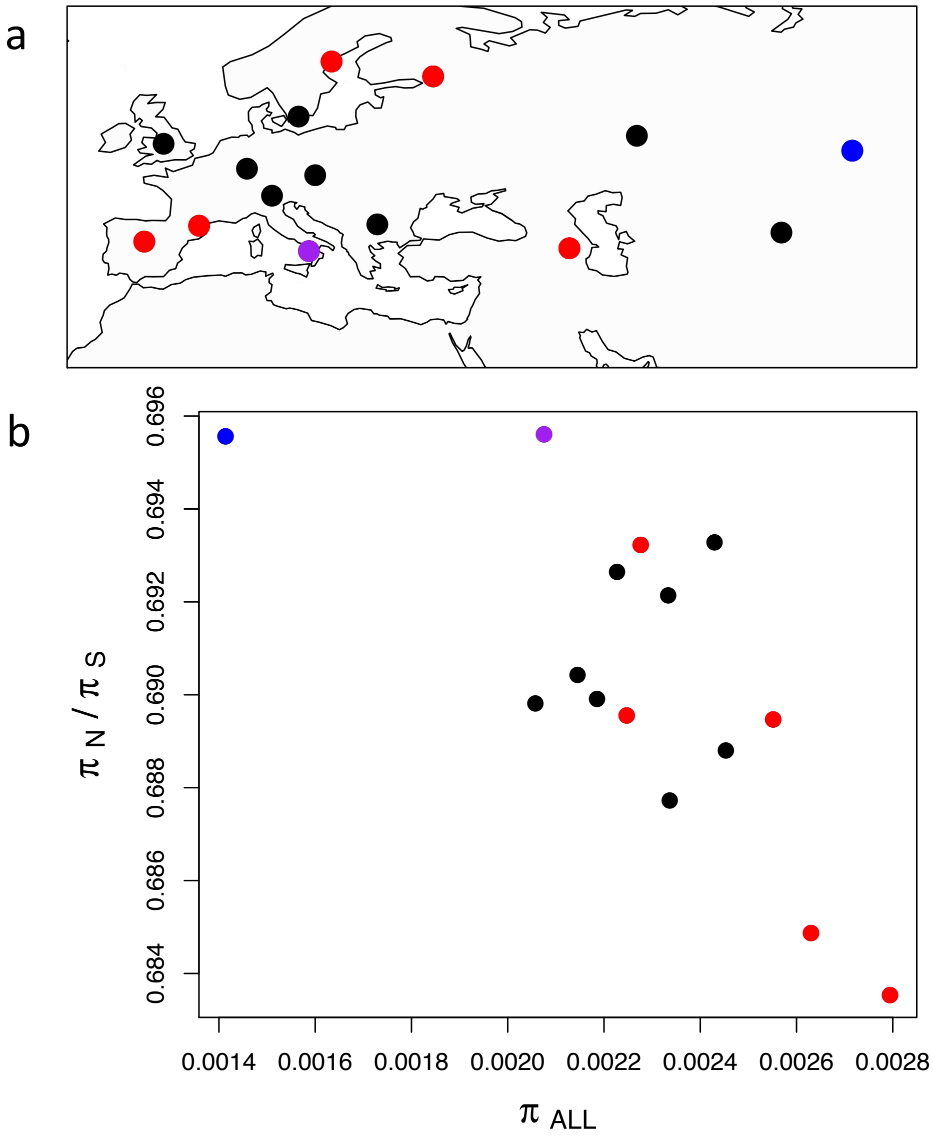

Supplementary Figure 6. Overall polymorphism (π_{ALL}) and the ratio of non-synonymous to synonymous polymorphism (π_N / π_S) in each geographical region. Blue and purple are two regions with highest π_N / π_S and low π_{ALL}, which do not overlap with regions with high amounts of relict haplotypes (red). The map was created with data from package "rworldmap" of R.

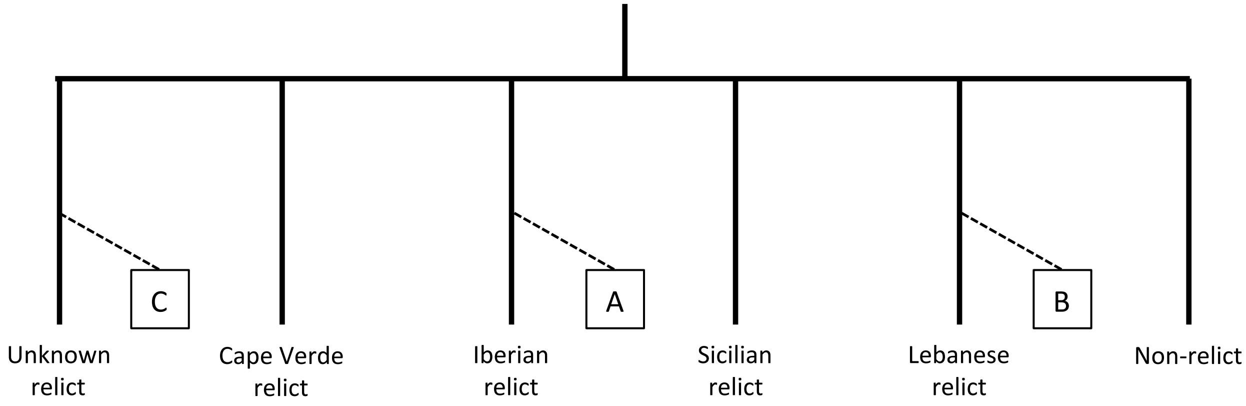

Supplementary Figure 7. A hypothetical phylogenetic tree in one 10-kb window, with non-relict, four known relicts, and one unknown relict. For an outlier haplotype identified in one individual (which we know does not belong to non-relict), we estimated its distance to all four known relict groups and assign the closest as its origin. For example, outlier haplotype A originated from Iberian relict and B from Lebanese relict. If, for example, outlier haplotype C's distance to any known relict is still larger than the threshold we used to detect outlier haplotypes, it came from an unknown relict group. We compared the proportion of outlier haplotypes with unknown origin among populations. Specifically, we asked whether northern populations have more outlier haplotypes with unknown origin than a southern reference population (Iberian non-relict) whose sources of outlier haplotypes are mostly known.

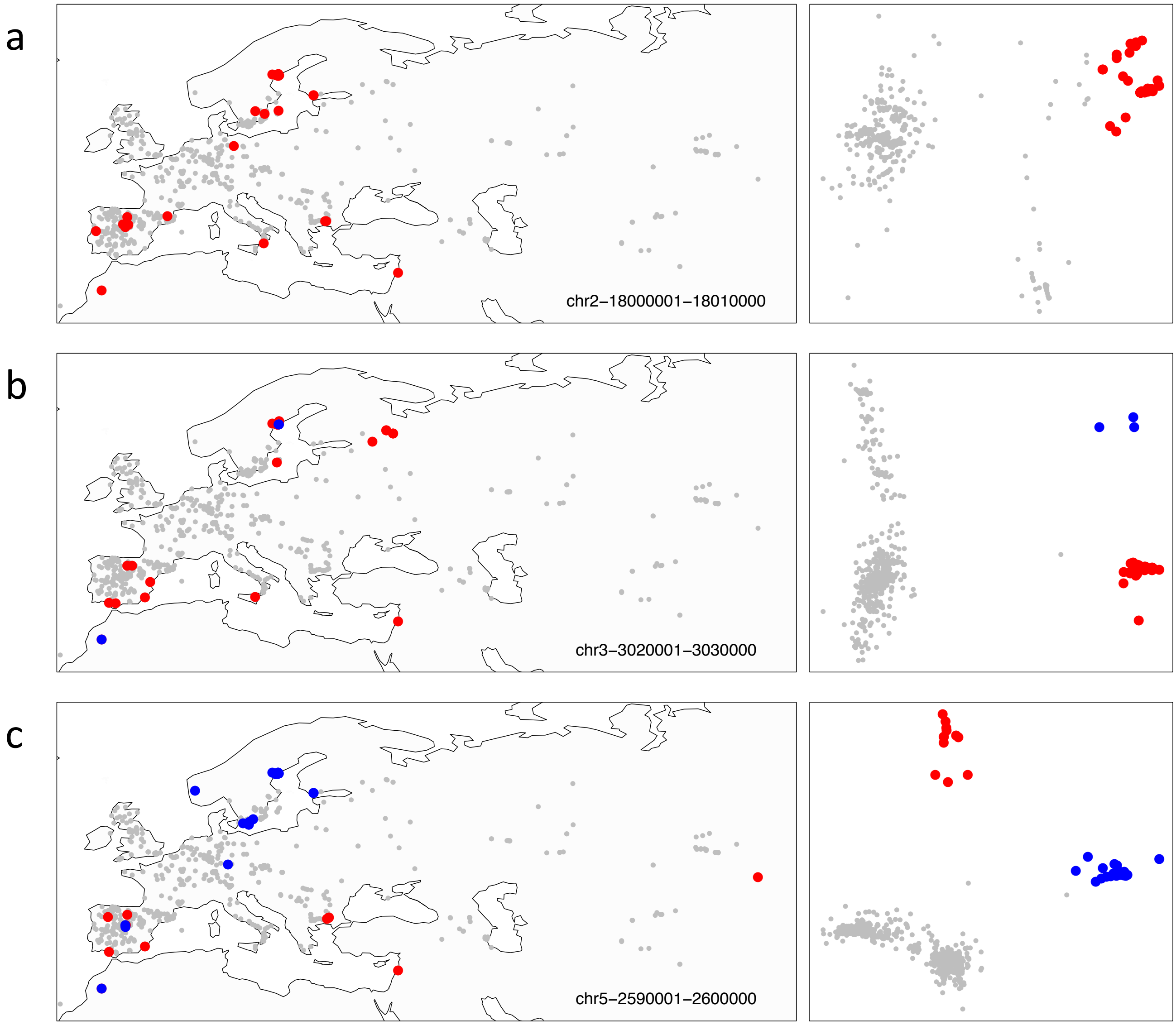

Supplementary Figure 8. Exemplary 10kb windows showing the disjunct distribution of outlier haplotypes and the southern origin of outlier haplotypes in the north. In each panel, on the left is the geographic distribution, and on the right is the principal coordinate analyses (PCoA) of the same accessions (horizontal: PCoA1, vertical: PCoA2). Grey dots are non-outliers, and colorful dots are outlier haplotypes. Blue or red colors are determined by whether the outlier haplotypes further separate into different groups based on PCoA. The maps were created with data from package "rworldmap" of R.

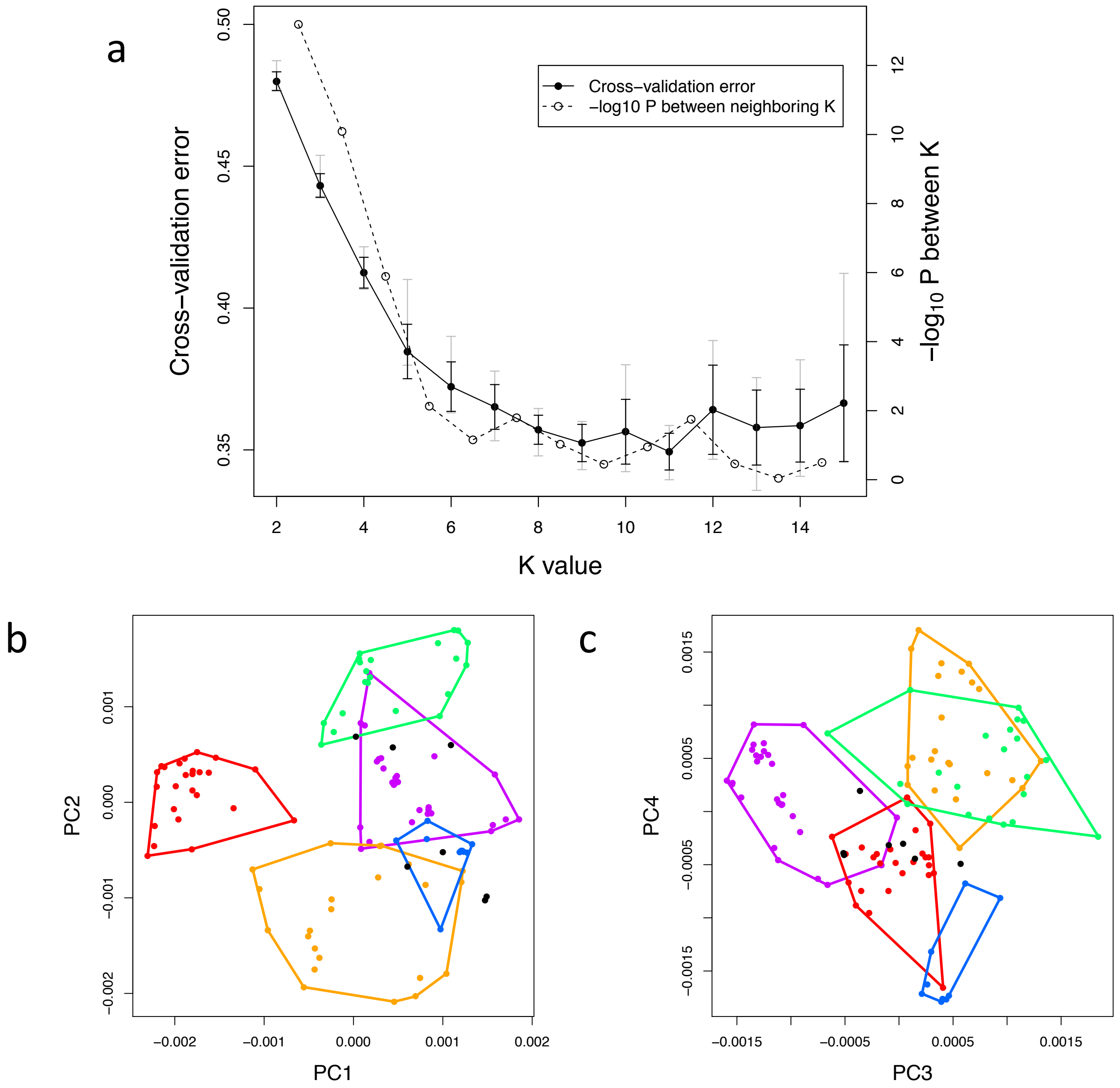

Supplementary Figure 9. ADMIXTURE cross-validation errors (panel **a**) and principal coordinate analyses (PCoA, panels **b-c**) among the ancestral haplotypes of chromosome 1 translocation. (**a**) For cross-validation error, vertical black lines denote mean +- one standard deviation and grey lines denote the range of cross-validation errors of 10 independent runs of each K value. -log10 *P* values were obtained from t-tests of cross-validation errors between neighboring K values. (**b-c**) Five colors represent five ADMIXTURE groups. Black dots represent admixed haplotypes whose ancestry do not exceed 0.5 in any ADMIXTURE group.

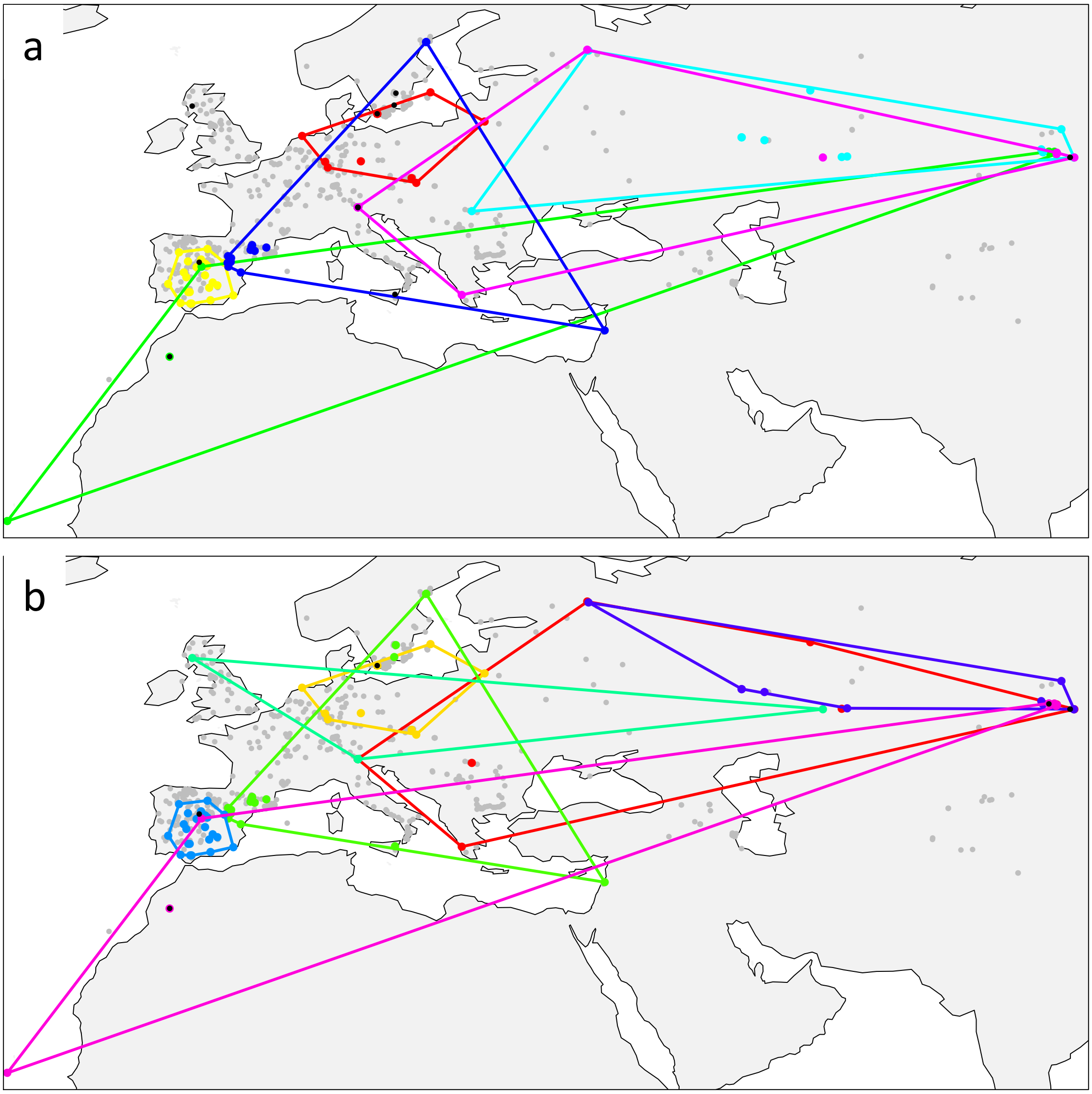

Supplementary Figure 10. Geographical distribution of accessions with the derived (grey dots) or ancestral (colored dots) haplotypes. For ancestral haplotypes, the two panels show K value equals 6 (**a**) and 7 (**b**). Black dots are admixed ancestral haplotypes. The maps were created with data from package "rworldmap" of R.
